# Carcinome adénoïde kystique tracheal

**Published:** 2010-12-21

**Authors:** Yassine Ouadnouni, Mohammed Bouchikh, Ahmed Jahid, Abdellah Achir, Mohamed Smahi, Yassine Msougar, Marouane Lakranbi, Leila Herrak, Najat Mahassini, Abdelatif Benosman

**Affiliations:** 1 Service de Chirurgie Thoracique CHU Ibn Sina, Rabat, Maroc,; 2 Laboratoire Central d’Anatomie et de Cytopathologie, CHU Ibn Sina, Rabat, Maroc

**Keywords:** Carcinome adénoïde kystique, chirurgie, trachée

## Abstract

**Abstract:**

Les carcinomes adénoïdes kystiques (CAK), ou cylindromes, sont des tumeurs malignes de la trachée qui représentent 0,1% des cancers des voies respiratoires. Il s'agissait d'une étude rétrospective à propos de quatre patients opérés dans notre service (deux femmes et deux hommes), ayant un âge moyen de 35,8; avec un suivi moyen de 7,2 plus ou moins 3,1 ans. Ils ont été admis pour tumeur trachéale se manifestant par une dyspnée inspiratoire. La bronchoscopie a montré une tumeur lisse de la trachée, dont la biopsie a révélé un CAK. Tous les malades ont bénéficié d’une résection de la tumeur avec recoupe trachéale et anastomose termino-terminale. La radiothérapie adjuvante a été indiquée dans un cas. L’évolution était marquée par la récidive locale avec des métastases pulmonaires après 6 ans chez un patient, et l'apparition des adénopathies cervicales métastatiques après 8 ans de suivi chez une autre. Les carcinomes adénoïdes kystiques sont des lésions de croissance lente, de découverte souvent tardive. La symptomatologie clinique est longtemps bien tolérée. Le traitement optimal est la chirurgie, la radiothérapie est indiquée quand la résection est incomplète ou impossible, ou après une récidive locale.

## Introduction

Les carcinomes adénoïdes kystiques (CAK), ou cylindromes, ont été décrits pour la première fois en 1873 par Billroth. Ce sont des tumeurs malignes de la trachée qui représentent 0,1 % des cancers des voies respiratoires. Ils sont la deuxième plus fréquente tumeur maligne primitive de la trachée.

## Méthodes

Il s'agit d'une étude rétrospective à propos de quatre patients opérés dans notre service sur la période de 1997 à 2009 avec un suivi moyen de 7,2 ans (plus ou moins 3,1 ans). Pour chaque patient, nous avons relevé: l'âge, le sexe, les antécédents, les signes fonctionnels, les signes cliniques, les données de la bronchoscopie et de la radiologie, le geste chirurgical, la description anatomopathologique de la pièce opératoire et le suivi.

## Résultats

Il s'agit de deux femmes et de deux hommes ayant un âge moyen de 35,8 ans (plus ou moins 7,3 ans) avec des extrêmes d'âge de 28 ans et 48 ans. Dans tous les cas, la tumeur est découverte à l’occasion de manifestations cliniques à type de dyspnée, toux chronique, hémoptysie et infections broncho-pulmonaires à répétitions (Tableau 1); avec un délai moyen de révélation de 17 mois (8 à 24 mois). L'état général des malades est conservé.

**Figure 1:**
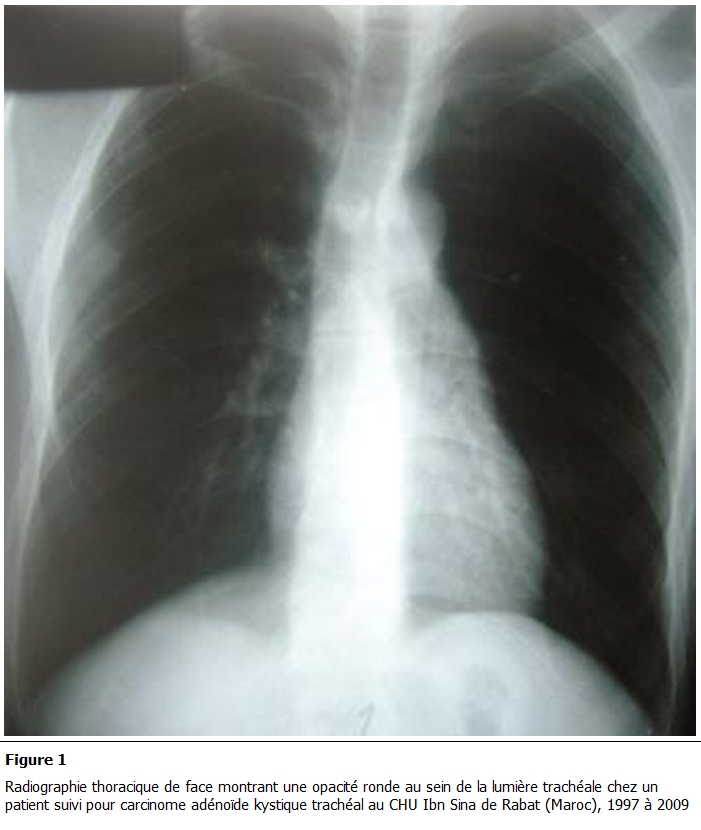
Radiographie thoracique de face montrant une opacité ronde au sein de la lumière trachéale chez un patient suivi pour carcinome adénoïde kystique trachéal au CHU Ibn Sina de Rabat (Maroc), 1997 é 2009

La radiographie thoracique a montré chez deux patients une opacité endotrachéale, dense, homogène, se confondant avec la paroi trachéale, et chez les deux autres un rétrécissement de la lumière trachéale (Figure 1).

**Figure 2:**
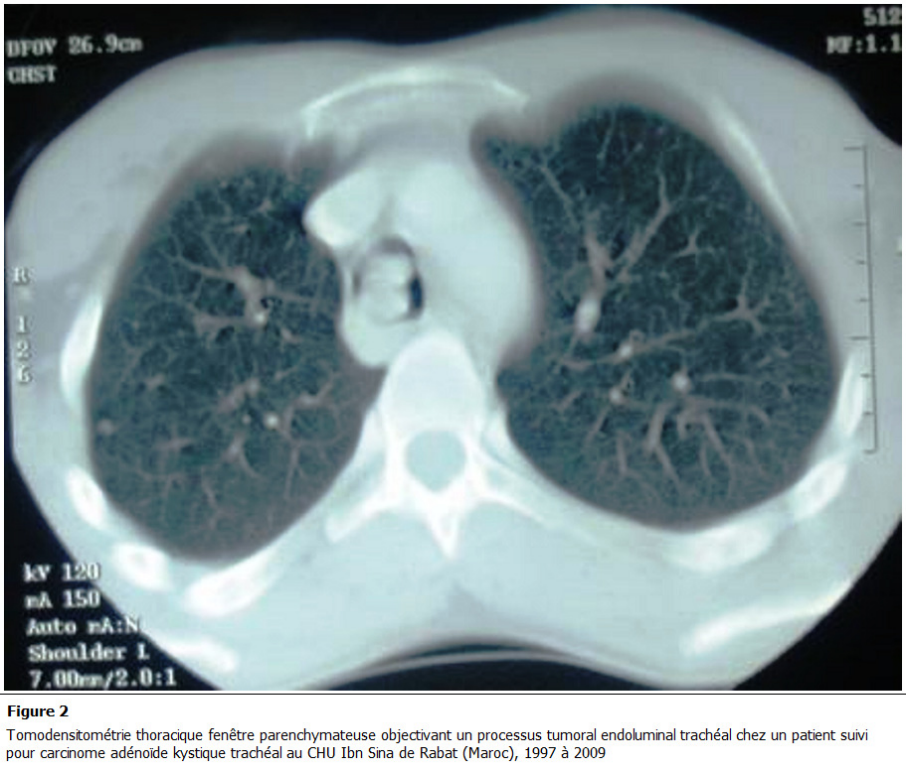
Tomodensitométrie thoracique fenêtre parenchymateuse objectivant un processus tumoral endoluminal trachéal chez un patient suivi pour carcinome adénoïde kystique trachéal au CHU Ibn Sina de Rabat (Maroc), 1997 é 2009

La tomodensitométrie a trouvé un processus tumoral endotrachéal se rehaussant de fa殮 hétérogène après injection, et a précisé son siège, son étendue et le caractère infiltratif ou non de la paroi par cette tumeur. Dans deux cas la tomodensitométrie a objectivé un développement exo mural de la tumeur, sans atteinte ganglionnaire (Figure 2). La bronchoscopie a visualisé la tumeur, et dont la biopsie a permis de confirmer le diagnostic de carcinome adénoïde kystique (Tableau 2).

**Figure 3:**
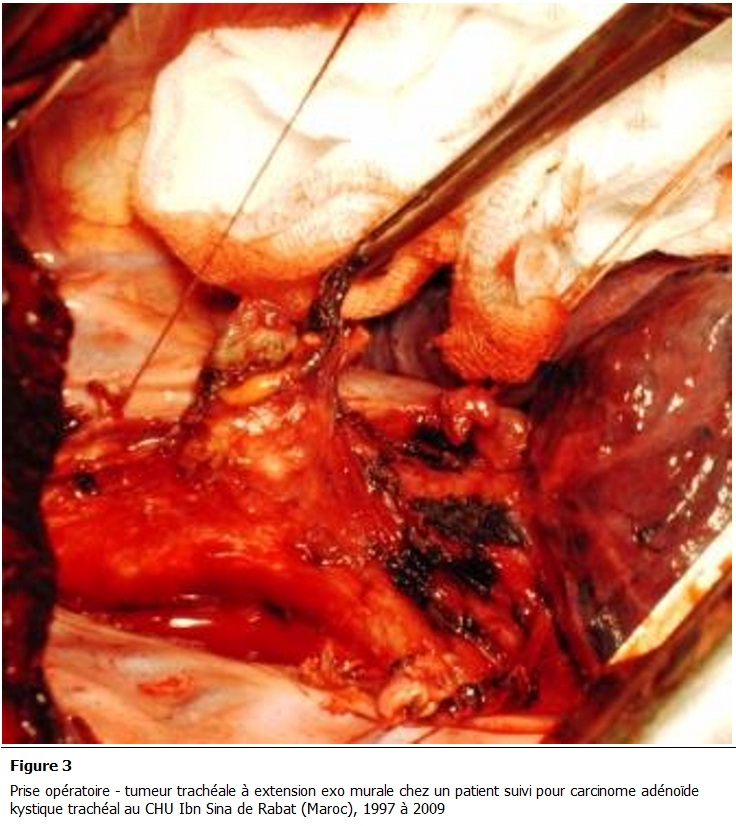
Prise opératoire - tumeur trachéale é extension exo murale chez un patient suivi pour carcinome adénoïde kystique trachéal au CHU Ibn Sina de Rabat (Maroc), 1997 é 2009

**Figure 4:**
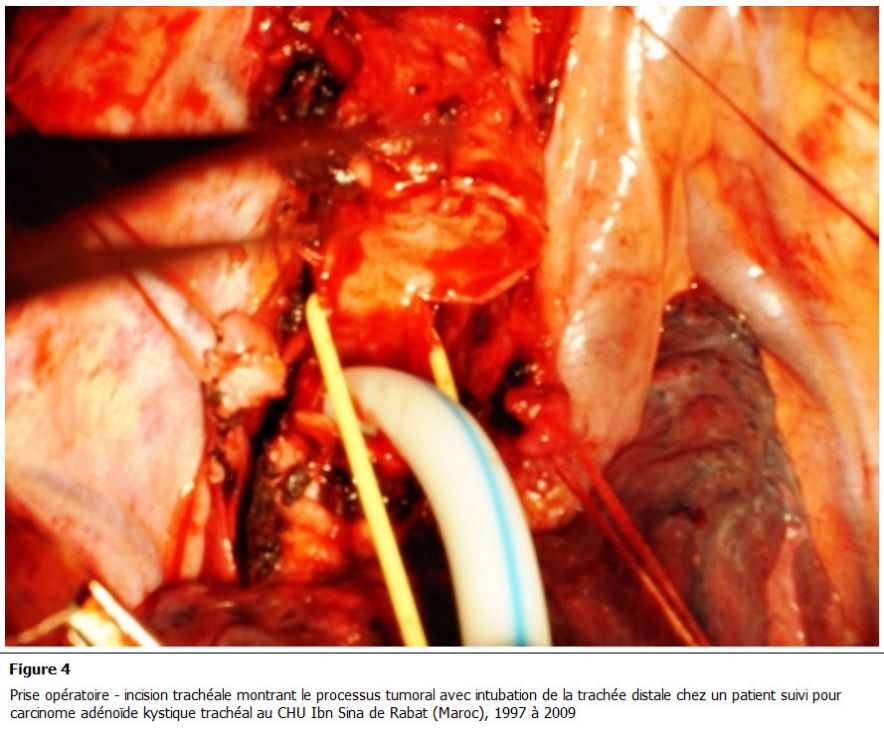
Prise opératoire - incision trachéale montrant le processus tumoral avec intubation de la trachée distale chez un patient suivi pour carcinome adénoïde kystique trachéal au CHU Ibn Sina de Rabat (Maroc), 1997 é 2009

**Figure 5:**
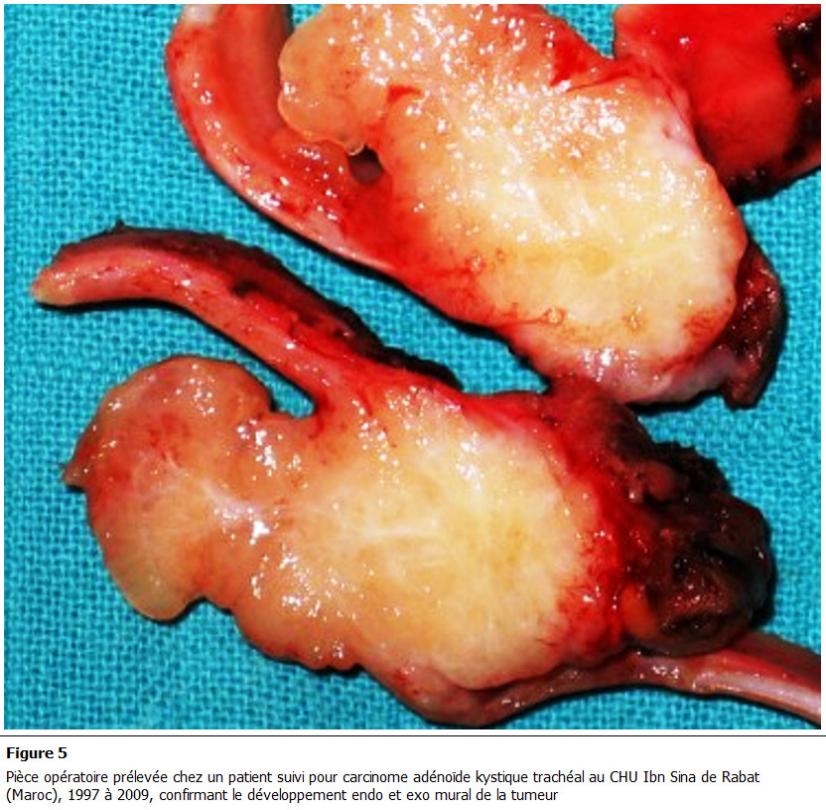
Pièce opératoire prélevée chez un patient suivi pour carcinome adénoïde kystique trachéal au CHU Ibn Sina de Rabat (Maroc), 1997 é 2009, confirmant le développement endo et exo mural de la tumeur

**Figure 6:**
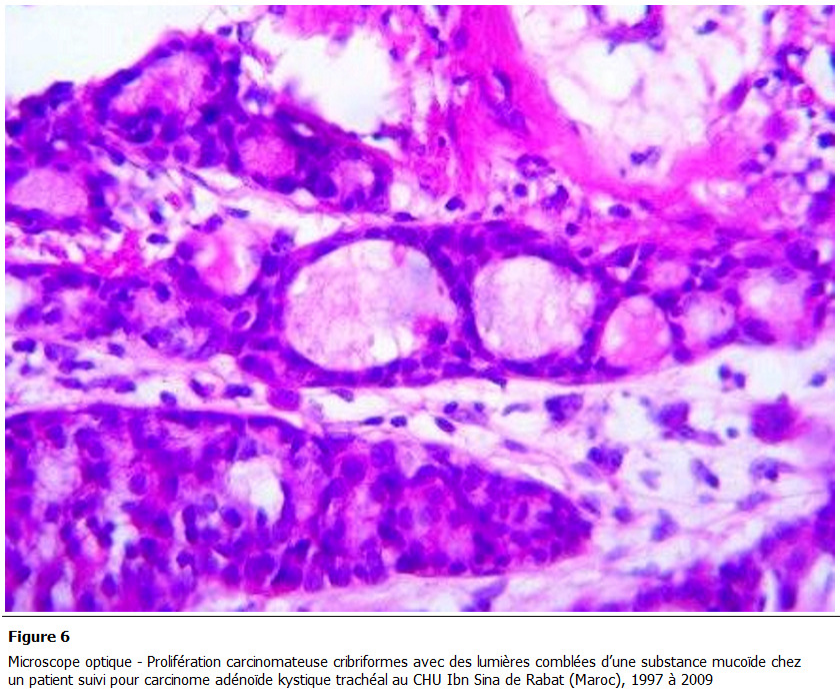
Microscope optique - Prolifération carcinomateuse cribriformes avec des lumières comblées d’une substance mucoïde chez un patient suivi pour carcinome adénoïde kystique trachéal au CHU Ibn Sina de Rabat (Maroc), 1997 é 2009

Tous nos malades ont bénéficié d’une résection chirurgicale. La voie d'abord réalisée est une cervico-sternotomie dans deux cas, une cervicotomie dans un cas et une thoracotomie postéro latérale droite passant par le 4éme espace intercostal dans un autre cas. Le geste a consisté en une résection complète de la tumeur emportant le segment trachéal d’implantation avec une recoupe trachéale puis une anastomose termino-terminale et curage ganglionnaire (Figure 3, 4). En aucun cas n'a été constaté un envahissement œsophagien ou vasculaire. La radiothérapie adjuvante a été indiquée dans un cas compte tenu d'un contact intime avec des éléments vasculaires. Les suites opératoires sont simples, on ne déplore aucun décès. L'étude histologique de la pièce opératoire met en évidence une prolifération carcinomateuse généralement cribriforme avec des lumières comblées d’une substance mucoïde ou hyaline (Figure 5). Ces structures sont tapissées de cellules aux cytoplasmes éosinophiles, hyperchromatiques, les mitoses étaient peu nombreuses; le stroma tumoral correspond à un tissu fibreux ponctué de quelques éléments inflammatoires. L’infiltration en profondeur atteignait les plans cartilagineux qu’elle dissociait largement pour infiltrer le reste de la tunique. En conclusion, il s’agit d’un carcinome adénoïde kystique, les ganglions prélevés étaient indemnes (Figure 6).

L’évolution était marquée par une récidive locale avec des métastases pulmonaires après 6 ans chez un patient, et l'apparition des adénopathies cervicales métastatiques après 8ans de suivi chez la patiente qui a reçu une radiothérapie adjuvante, et pour lesquels une radio-chimiothérapie est proposée avec une désobstruction par photo résection endoscopique au laser pour la récidive locale.

## Discussion

Les carcinomes adénoïdes kystiques, anciennement appelés cylindromes, sont des tumeurs malignes épithéliales se développant le plus souvent aux dépens des glandes salivaires principales et accessoires. D’autres localisations tel que, les glandes de la muqueuse bronchique, ont été décrites [1]. Ces tumeurs s’observent surtout à un âge plus jeune que celui des autres cancers, sans prédominance de sexe, ni imputabilité du tabac. Les carcinomes adénoïdes kystiques sont souvent de diagnostic tardif, en effet la symptomatologie clinique est souvent très modérée et trompeuse, faisant évoquer plus un asthme, la manifestation principale est une dyspnée. Cette symptomatologie clinique est longtemps bien tolérée du fait du large calibre de la trachée et de la croissance lente de la tumeur. Le délai moyen de révélation après le premier symptôme est souvent supérieur à six mois [1,2].

Dans notre étude, nos données sont comparables à celle de la littérature. Nos patients sont jeunes avec un âge moyen de 35 ans, les deux sexes sont représentés de manière égale, aucune habitude toxique n'est retrouvée. Le signe fonctionnel principal est la dyspnée, avec un délai moyen de révélation de 17 mois.

La radiographie thoracique peut sembler normale ou montrer une opacité latéro-trachéale, ou bien une tumeur endotrachéale. La tomodensitométrie thoracique permet d’évaluer l’extension péri trachéale de la tumeur et de découvrir une éventuelle lésion secondaire pulmonaire ou ganglionnaire. La fibroscopie trachéobronchique est indispensable, elle permet de préciser le siège et de confirmer le diagnostic en effectuant une biopsie qui est souvent de lecture difficile [2].

Le traitement repose sur trois modalités thérapeutiques, qui sont la chirurgie, la radiothérapie, et l’endoscopie interventionnelle. La chimiothérapie n’a pas de place en dehors des formes métastasiques [3,4]. La chirurgie consiste en une résection tumorale avec recoupe trachéale et anastomose termino-terminale puis curage ganglionnaire satellite. Cette chirurgie est délicate et nécessite une équipe chirurgicale et anesthésique entraîn#x000E9;e. L’étendue en hauteur de la résection rend le plus souvent l’anastomose difficile, elle est la source de complication postopératoire ou de récidive. Le curage ganglionnaire ne doit pas être trop extensif afin de ne pas compromettre la vascularisation trachéale. La voie d’abord diffère selon le siège, pour une meilleure approche on peut être amené à réaliser une cervicotomie pour les localisations hautes, soit une cervicosternotomie pour la jonction tiers-moyen tiers-inférieur, soit une thoracotomie postéro latérale passant par le 4e espace intercostal pour les localisations proches de la carène. Le taux de mortalité péri opératoire varie selon les séries entre 9 et 13% [3,4].

Tous nos malades ont été opérés, la voie d’abord est dictée par la localisation, la résection chirurgicale est jugée satisfaisante. Une radiothérapie adjuvante est recommandée, à une dose variant de 45 à 65 Gray selon les équipes, certains préconisent systématiquement une radiothérapie, alors que d’autres ne proposent ce traitement que lorsque les tranches de section sont envahies. Un délai d’un mois est, au moins, recommandé après la chirurgie et il peut être utile de réaliser une fibroscopie bronchique afin de s’assurer de la cicatrisation. Une radiothérapie exclusive est proposée à une dose supérieure à 60 Gray lorsque la lésion est considérée irrésécable. La curiethérapie endobronchique peut être utilisée pour augmenter la dose totale d’irradiation et améliorer le taux de contrôle local [3,4].

Dans notre approche la radiothérapie est indiquée quand la résection est incomplète ou impossible ou après une récidive locale et en cas d’envahissement ganglionnaire. Dans le cas d’une obstruction tumorale sévère, la photo résection endoscopique par laser permet rapidement une désobstruction, et qui sera éventuellement suivie d’un traitement local optimal [5].

Une surveillance rapprochée et au long cours endoscopique et radiologique est nécessaire afin de guetter toute récidive locale ou survenue de métastases qui sont souvent pulmonaire, hépatique, ganglionnaire et osseuse.

Le pronostic du carcinome adénoïde kystique est meilleur par rapport à celui du carcinome épidermoïde avec un taux de survie à 5 ans et à 10 ans de 75 % et de 50% respectivement. La médiane de survie selon Grillo et al, est de 118 mois après résection complète, est de 90 mois après résection incomplète associé à une radiothérapie, est de 28 mois après une radiothérapie exclusive [5].

## Conclusion

Le carcinome adénoïde kystique de la trachée est une tumeur rare. Son diagnostic et sa surveillance repose sur la fibroscopie bronchique associée à la biopsie. La tomodensitométrie évalue mieux l’étendue et l’extension péri trachéale de cette tumeur. La prise en charge thérapeutique repose essentiellement sur la chirurgie couplée à la radiothérapie.

## Conflit d’intérêts

Les auteurs ne déclarent aucun conflit d’intérêts.

## Consent

Les auteurs déclarent avoir reçu le consentement des patientes pour reporter cette série.

## Contribution des auteurs

Tous les auteurs ont participé à la prise en charge du patient et à la rédaction du manuscrit.

## Figures and Tables

**Tableau 1:** Les signes fonctionnels révélateurs de carcinome adénoïde kystique trachéal chez quatre patients suivis au CHU Ibn Sina de Rabat (Maroc), de 1997 à 2009

**Symptômes**	**n**	**%**
Dyspnée	4	100
Toux	3	75
Hémoptysie	3	75
Infection BP à répétition	2	50

BP: Broncho-pulmonaire, n : nombre

**Tableau 2:** L'aspect bronchoscopique de carcinome adénoïde kystique trachéal chez quatre patients suivis au CHU Ibn Sina de Rabat (Maroc), de 1997 à 2009

**Patient**	**siège**	**aspect**
1	1/3 moyen –supérieur	3 bourgeons lisse; pédiculé
2	1/3 moyen	lisse
3	1/3 moyen	lumière réduite à moins 30%
4	1/3 inférieur	lisse; sessile; lumière réduite à moins 20%
